# Asymmetrically Spatial Effects of Urban Scale and Agglomeration on Haze Pollution in China

**DOI:** 10.3390/ijerph16244936

**Published:** 2019-12-05

**Authors:** Qingyu Fan, Shan Yang, Shuaibin Liu

**Affiliations:** 1 School of Geographic Science, Nanjing Normal University, Nanjing 210023, China; qingyufqy@126.com; 2 Jiangsu Center for Collaborative Innovation in Geographical Information Resource Development and Application, Nanjing 210023, China; 3 School of Civil Architecture, Zhengzhou University of Aeronautics, Zhengzhou 450000, China; liushuaibin@zua.edu.cn

**Keywords:** urban scale, urban agglomeration, haze pollution, China

## Abstract

Rapid urbanization in China not only promotes the rapid expansion of urban population and economic agglomeration, but also causes the aggravation of haze pollution. In order to better clarify the asymmetric and nonlinear effects of urban scale and agglomeration on haze pollution, this paper quantitatively evaluates the spatial spillover effects of population size and economic agglomeration on haze pollution in 342 Chinese cities from 2001 to 2016 by using exploratory spatial data analysis (ESDA) and spatial econometric model. The results show the following: (1) During the research period, the distribution of urban scale, agglomeration, and haze pollution in China presented complex asymmetrical features, with the former two presenting a “core–periphery” distribution mode, while the latter having a tendency to spread around. In addition, under the influence of urban population size and economic agglomeration, haze pollution in Chinese cities presents significant spatial autocorrelation, with the agglomeration degrees showing a fluctuating upward trend during the study period. (2) Both urban scale and urban agglomeration have positive global spatiotemporal correlation with haze pollution. Local spatial correlation features are more obvious in China’s emerging urban agglomerations like Beijing–Tianjin–Hebei and Yangtze River Delta. (3) The spatial effects of haze pollution are better evaluated by spatial Durbin model (SDM) with spatial fixed effects, obtaining a coefficient of 0.416, indicating haze in neighboring cities affected each other and had significant spillover. By decomposing the effect of urban scale and agglomeration on haze as direct and indirect effects, the direct effect of urban population size and the indirect effect of urban economic agglomeration are found to be more prominent, reflecting that significant asymmetrical characteristics exist in the spatial effects of urban size and agglomeration on urban haze. (4) Among the control variables that affect China’s rapid urbanization, the level of urban economic development has a positive effect on haze pollution, while the high-level industrial structure and improved technical level can effectively reduce haze pollution. Continuous decline of haze concentration of Chinese cities in recent years has been indicating the spatial relationships between haze and urban size and agglomeration have a decoupling trend. The findings contribute to theory by emphasizing the spillover effect and spatial heterogeneities of geographical factors, and have implications for policy makers to deal with haze pollution reasonably and effectively.

## 1. Introduction

With the rapid development of urbanization, industrialization, and modernization, great changes have taken place in economy, society, resources, and environment, among which the air quality and human health problems caused by haze has attracted much attention all over the world, especially in developing countries [1,2,3,4]. The rapid economic growth of China in recent decades has resulted in serious air pollution problems on both local and regional scales [5,6,7]. Megacities in China such as Beijing, Tianjin, and Shanghai have suffered from haze episodes frequently with the daily mass concentrations of fine particulate matter (PM_2.5_, fine particulates with aerodynamic diameter less than 2.5 μm) over the Chinese air pollution standard of 75 μg/m^3^ (China National Environmental Monitoring Centre, 2013), which is three times higher than the air quality guideline of 25 μg/m^3^ recommended by the World Health Organization (WHO) [5,8,9]. In 2013, a report entitled “Towards an Environmentally Sustainable Future: Country Environmental Analysis of the People’s Republic of China” pointed out that seven of the 10 most polluted cities in the world were located in China (Taiyuan, Beijing, Urumqi, Lanzhou, Chongqing, Jinan, and Shijiazhuang), and less than 1% of China’s 500 large cities met the air quality standards proposed by WHO [10]. Therefore, most Chinese cities have been taking many efforts to improve air quality, such as guiding the orderly flow of population, adjusting the industrial structure, improving the quality of economic development, and optimizing the field of foreign investment. In recent years, China’s great efforts that have been made to control haze and the fact that PM_2.5_ concentration has been significantly declined were unprecedented in the world history of haze control [11,12,13].

Although haze weather has some relation with natural conditions, the fundamental reasons are still the growth of urban population scale and economic agglomeration (the phenomenon that large numbers of economic units and activities are concentrated in a relatively limited space), as well as the long-term irrational energy structure and industrial structure [8,14,15,16]. For example, mid-eastern China with more severe haze pollution is also a region with large size of urban population and highly concentrated industries [6,17,18]. From the perspective of China’s haze aggravation process, haze is closely related to the rapid urbanization and industrialization [14,18,19]. Large-scale manufacturing production, continuous infrastructure construction, and high-density automobile exhaust emissions have significantly increased the concentration of PM_2.5_ in the air [20,21]. Given the continuous expansion of urban population and the increasing degree of economic agglomeration, will the haze continue to worsen? How much has the urban population size and economic agglomeration contributed to the haze pollution and what is the difference between their effects? In the process of urbanization influenced by such factors as economic development level, industrial structure adjustment, technological innovation, and foreign investment, what role do they play respectively in the generation and change of haze? The emergence and solution of these questions has become an important scientific issue in the field of geography and sustainable development. The emergence and solution of these questions, which are important scientific topics in the field of geography and sustainable development, is the starting point and focus of this study, respectively.

In the 21st century, China’s rapid urbanization has been becoming a major event with global effects, bringing a non-negligible influence on the air quality closely related to human beings [1,22]. Therefore, in order to obtain a clearer knowledge of the asymmetric and nonlinear responses of air pollution to the urbanization factors including urban scale and urban agglomeration, we explore the spatial and temporal distribution and correlation patterns between them and their spatial spillover effects and interactions. Through this study, we aim to fill the knowledge gap by contrastive and quantitative spatial analysis of the impacts of urban population size and urban economic agglomeration on haze pollution through spatial econometric models. Furthermore, we hope this research can have implications for national and local policy-makers in China and other countries in the world to address the air pollution and provide theoretical reference for scientifically and accurately preventing and controlling haze. The remainder of this paper is structured as follows: Section 2 is the Literature Review. Section 3 introduces the methods and data used in this paper, followed by Section 4—Results, which presents the spatiotemporal distribution, spatial correlation, and spatial effects of urban scale, agglomeration, and haze pollution. Section 5 is the Discussion about our findings. The last section is the Conclusion and Policy Implications of this research.

## 2. Literature Review

As a state of air pollution, haze is suffered from by countries all over the world to different degrees in the process of urbanization and industrialization. Thus, lots of scholars and organizations have carried out a large number of studies on the distribution and causes of haze pollution; the components of haze are complex and diverse [23,24,25,26]. According to relevant literature, PM_2.5_ is the primary pollutant of haze whose components are extremely complex and diverse, mainly including SO_2_, nitrogen oxides (NO_x_), and respirable particulate matter (PM), which consists of matters with aerodynamic equivalent diameter less than or equal to 2.5 microns in the air [5,6,27]. These inhalable particles have a major impact on human health and increase the incidence of cardiovascular and respiratory diseases, which lead to higher mortality among vulnerable populations [28,29,30]. Based on the existing research literature, PM_2.5_ concentration is mainly related to the urban population size and economic agglomeration on the macro scale. For the spatial distribution of PM_2.5_, the literature has focused on understanding the temporal change and spatial diffusion of PM_2.5_ [31,32,33,34]. Based on the atmospheric PM_2.5_ monitoring results of Nanjing, an empirical literature concluded that PM_2.5_ concentration is related to the location of city and its transportation process, and interacts with the surrounding cities [35]. For the impact of urban population size and urban economic agglomeration on haze, past literature has shown that the rapid agglomeration of population is an important reason for the aggravation of haze. The mechanism is that the expansion of urban scale aggravates urban sprawl and increases residents’ commuting distance, leading to the mass use of automobiles and thus increasing the concentration of PM_2.5_ in the atmosphere of each city [3,14]. Moreover, the gathering of economic units and activities in big cities strengthens the concentration of PM_2.5_ emitted by industrial and other related enterprises [36,37]. However, there exist some scholars who deem that urbanization can significantly improve the utilization efficiency of energy and resources, and urban economic agglomeration is conducive to the alleviation of haze pollution [37,38,39]. In addition, the concept of “Environmental Kuznets Curve” was proposed to hold that when a country or region is at a low economic development level, its environmental quality will deteriorate with economic growth. Nevertheless, when its economic development reaches a certain level, the environment quality will begin to improve with economic growth [39]. Based on this concept, an empirical study of China as the case found that that industrial agglomeration in 31 Chinese provinces can intensify the degree of haze pollution [13]. China’s economic agglomeration is changing from labor-intensive to knowledge-intensive and service-intensive [10,17].

With the gradual opening of the international haze monitoring data in recent years, literature on the influencing factors of haze and its countermeasures from the perspective of spatial econometrics has gradually increased [14,19,20]. Relevant research analyzing the correlation between the haze pollution and economic growth in China found that their relationship presented a significantly positive U-shaped trend [17]. Another study drew such a conclusion that haze pollution was not only affected by economic growth, population size, and urbanization, but also by the haze pollution in the previous period [18,19,20,40]. They all provided evidence for the study of a nonlinear relationship between urban scale and agglomeration and haze pollution. As a product in the early stage of urbanization and industrialization, haze and its relationship with urban population scale and economic agglomeration are very complex. Hence, there are still great differences in the conclusions of scholars from different countries and research backgrounds, which may be due to the great differences of natural conditions in their study areas [4,14,41]. In addition, the existence of an asymmetric effect of urban scale and agglomeration on haze pollution and the nonlinear characteristics formed by their dynamic relationship may also be the causes of this difference.

Considering that a large number of studies have confirmed the major impact of urban scale and agglomeration on haze pollution, coupled with the significant spatial coupling characteristics and intuitive phenomena of haze pollution and urban scale and agglomeration in Chinese urban scale, thus it is urgent to include these indicators of urban population size and economic agglomeration as explanatory variables in the spatial econometric models to quantify and contrast their effect on haze pollution. Furthermore, there are still some limitations on the spatial and temporal scale in the existing literature, whose methods are mainly simple linear correlation analyses, because they usually neglect the existence of spatial effect and also fail to pay much attention to the spatial interaction and marginal effects of explanatory variables [33,37]. To fill this knowledge gap, therefore, we explore the systematically spatiotemporal distribution and correlation of urban population size, economic agglomeration, and haze pollution (represented by PM_2.5_ concentration) of 342 Chinese cities from 2001 to 2016, and quantitatively analyze their spatial effect by adopting exploratory spatial data analysis and spatial econometric models. Through this research, we aim to provide a more comprehensive understanding of the spatial and temporal dynamics differentiation of urban scale, agglomeration, and haze and their asymmetrical relationship and interaction mechanisms. Indeed, it will be more meaningful for our research to provide some useful reference for local governments to implement effective haze management measures.

## 3. Methodology and Variables Selection

### 3.1. Methodology

#### 3.1.1. Exploratory Spatial Data Analysis

ESDA based on Geographic Information System (GIS) is a spatial statistics method with spatial autocorrelation measurement as the core, which can effectively reveal the spatial distribution characteristics and laws of geographical elements [42,43]. Spatial autocorrelation includes global spatial autocorrelation and local spatial autocorrelation. Generally, there is only one variable in the global spatial autocorrelation analysis, while the global spatial autocorrelation analysis measured by Bivariate Moran’s I has higher applicability and validity in measuring two spatial variables’ spatial correlation and dependence. The Bivariate Moran’s I can be defined as follows [42]:

(1) $$I=\sum_{i=1}^{n}\sum_{j\neq i}^{n}w_{ij}(x_{i}-\bar{x})(y_{j}-\bar{y})/s^{2}\sum_{i=1}^{n}\sum_{j\neq i}^{n}w_{ij}$$

where *I* is the indicator of bivariate global spatial autocorrelation. The larger the *I* value, the greater the spatial correlation between PM_2.5_ and urban scale or agglomeration. *x_i_* and *y_j_* are the values of different observed variables of cities *i* and *j*. *n* is the number of all cities. *s*^2^ is the sample variance. *w_ij_* is the spatial weight matrix constructed by neighborhood criteria to measure spillover effect, which is designed such that *w_ij_* = 1 if city *i* and city *j* are closer to each other than any other city, otherwise *w_ij_* = 0 [30].

The Bivariate Local Moran’s *I* is defined as follows [42]:

(2) $$I_{i}=\frac{y_{i}-\bar{y}}{s^{2}}\sum_{i=1,j\neq i}^{n}w_{ij}(y_{j}-\bar{y})$$

where *I_i_* is the indicator representing the local correlation between PM_2.5_ concentration in city *i* and urban scale or agglomeration of city *j*, which can be divided into four different types of HH (high–high), HL (high–low), LH (low–high), and LL (low–low). HH and LL represent that the average annual concentration of PM_2.5_ concentration in city *i* is positively spatially correlated with the urban scale or agglomeration of city *j*, that is, cities with high (low) PM_2.5_ concentration are surrounded by cities with large (small) population size or high (low) economic agglomeration. While HL and LH indicate PM_2.5_ concentration in city *i* is negatively spatially correlated with the urban scale or agglomeration of city *j*. All the above indicators are calculated by using the multivariable LISA in Open Geoda [42].

#### 3.1.2. Spatial Econometric Model

Traditional econometric models based on linear correlation invariably neglect the spatial position of observed objects, so they cannot reflect the spatial effect of geographical elements [44]. After the spatial autocorrelation analysis confirms the spatial dependence of geographical elements, therefore, it is necessary to establish a spatial econometric model to evaluate the spatial effect and interaction of geographical elements [6,45]. The commonly used spatial econometric models include spatial error model (SEM), spatial lag model (SLM) and spatial durbin model (SDM). Since SDM contains spatial dependent effects of both independent variables and dependent variables, it is more general and has more effective explanatory ability than SLM and SEM. Therefore, we adopted SDM to estimate the spatial effect of urban scale and agglomeration on haze pollution in 342 Chinese cities. The SDM is constructed as follows [46]:

(3) $$y_{it}=\delta \sum_{j=1}^{n}w_{ij}y_{jt}+\alpha x_{it}+\beta \sum_{j=1}^{n}w_{ij}x_{jt}+\mu_{i}+\nu_{t}+\varepsilon_{it}$$

where *y_it_* and *x_it_* represents the PM_2.5_ concentration value of city *i* and the attribute values of its independent variable at year *t*, respectively. *w_ij_* is the spatial weight matrix based on neighborhood criteria, which is the same as Equation (1). *δ* is the spatial lag coefficient which measures the magnitude of spillover effects in haze pollution, the values of which can be compared to identify the regional differences in spatial spillover effects based on the estimation results of subsamples from different regions. *α* and *β* are the regression coefficient vector and the spatial interaction coefficient vector of all the independent variables including core independent variables and control variables, respectively. In this proposed model, the observed values of all variables were logarithmized. Therefore, the estimated coefficients *α* and *β* can be interpreted as the elasticities of corresponding variables. *μ_i_* and *ν_t_* are the spatial and temporal effects, capturing time-constant unobservable variables of city *i* and space-constant unobservable variables of time *t,* respectively. *ε_it_* is a space error term that is independently identically distributed. If *β* = 0, *δ* ≠ 0, and SDM can be simplified to SLM. If *β + αδ* = 0, SDM can be simplified to SEM [6,46].

According to relevant literature, partial differentiation is required to decompose the spatial effect coefficients obtained by SDM [6,46]. The SDM could be converted into:

(4) $$Y={\left(I-\delta W\right)}^{-1}c\lambda_{N}+{\left(I-\delta W\right)}^{-1}(X^{\prime }\alpha +WX^{\prime }\alpha )+{\left(I-\delta W\right)}^{-1}\varepsilon$$

where *Y* is the vector of *N* × 1-dimensional dependent variable. *W* is an *N* × *N* spatial weight matrix. *λ_N_* is the *N* × 1-dimensional vector with all elements being 1. *c* is a constant term. *X’* is the *N* × *K-*dimensional matrix of all independent variables. *ε* represents the error term. The partial differential matrix *Y* is defined as follows [44]:

(5) $$\left[\frac{\partial Y_{i}}{\partial X_{1k}}\cdots \frac{\partial Y_{i}}{\partial X_{Nk}}\right]=\left[\begin{array}{ccc} \frac{\partial Y_{i}}{\partial X_{1k}} & \cdots & \frac{\partial Y_{1}}{\partial X_{Nk}}\\ \vdots & \ddots & \vdots \\ \frac{\partial Y_{N}}{\partial X_{1k}} & \cdots & \frac{\partial Y_{N}}{\partial X_{Nk}} \end{array}\right]={\left(I-\delta W\right)}^{-1}\left[\begin{array}{cccc} \alpha_{k} & w_{12}\theta_{k} & \cdots & w_{1N}\theta_{k}\\ w_{21}\theta_{k} & \alpha_{k} & \cdots & w_{2N}\theta_{k}\\ \vdots & \vdots & \ddots & \vdots \\ w_{N1}\theta_{k} & w_{N2}\theta_{k} & \cdots & \alpha_{k} \end{array}\right]$$

where *α_k_* is the direct effect, indicating the elasticity of the local city’s independent variables (urban scale, agglomeration, and other control variables) to dependent variables (haze pollution). *θ* is the influence coefficient of the independent variable of the neighboring city *j* on the dependent variable of the local city *i*, which is obtained by partial differentiation. The indirect effect is the mean of the elements except *α_k_* (such as *w*_12_*θ_k_*, *w*_1*N*_*θ_k_*, *w*_21_*θ_k_*, and so on) in the right-most matrix of the formula above, indicating the elasticity of the neighboring city’s independent variables to the dependent variables of the local city, which can also be called the spillover effect [44].

### 3.2. Variables Selection and Indicator Description

According to previous literature [17,47,48,49], haze pollution is not only spatially affected by urban scale and agglomeration, but also by many other factors related to urbanization, such as industrial structure, scientific and technological level, foreign direct investment, and so forth. Because this paper aims to discuss the asymmetrically spatial effect of urban scale and agglomeration on haze pollution, we took the haze pollution as the dependent variable. Urban scale and urban agglomeration were taken as the core independent variables into the proposed model. In addition, factors of foreign direct investment, scientific and technological level, and so on were taken as the control variables into the regression model [19,50,51]. These variables are specified as follows:

Dependent variable is haze pollution (*Pm*), which is represented by PM_2.5_ concentration (ug/m^3^). The PM_2.5_ data of 2001–2016 were obtained from the global PM_2.5_ gridded data sets at 0.01 degrees published by the Socioeconomic Data and Applications Center (SEDAC), which is accurate and applicable to reflect the change of haze pollution in China shown by relevant studies [6,52,53,54]. ArcGIS platform was used to vectorize this raster data into specific annual average concentrations of PM_2.5_ in each city. Since China began to publish its own PM_2.5_ data of monitoring stations in 2012, there is no available monitoring data to study China’s haze pollution of 2001–2016 [6].

Core independent variables composed of urban scale and agglomeration are specified as below:

- Urban scale (*Us*) is characterized by urban population size, measured by the total population at the end of each year in one city [37,39]. Excessive urban population size will bring more resource consumption, housing demand, and travel demand, which indirectly aggravates haze. Thus, *Us* is expected to be positive for haze pollution [54,55,56].
- Urban agglomeration (*Ua*) refers to urban economic agglomeration, which is represented by gross national product (GDP) at each year of one city. In the urbanization process, urban economic agglomeration is the result of the concentration of economic units and activities to cities, which will lead to the decline of air quality. Urban economic agglomeration includes industrial, capital, and labor agglomeration, which can be largely reflected by GDP of one city. Therefore, GDP is used to characterize the positive impact of urban economic agglomeration on haze pollution [15,37,51].

Control variables in the proposed model are specified as below:

- Per capita GDP (*Pgdp*) is represented by the per capita GDP of each city, which is obtained by deflating with the GDP index of 2001 as the base year [56,57]. Based on existing literature, *Pgdp* is expected to be positive for haze pollution [54,57].
- Advanced industrial structure (*Str*) is obtained by concluding the ratio of the output value of the tertiary industry to the output value of the secondary industry in one city [13,58]. As an important indicator of energy conservation and pollutant emission reduction, it reflects the degree of transformation of industrial structure from labor and capital intensive to technology and knowledge intensive. It is known that the secondary industry is the main source of industrial waste and air pollution, while the tertiary industry (including information technology, financial service, etc.) could reduce the negative externalities of environmental pollution [10]. Therefore, advanced industrial structure is chosen to reflect its negative impact on haze pollution.
- Scientific and technological level (*Tec*) is represented by the proportion of science and technology expenditure in the local financial budget to the GDP of the year [19,47]. To some extent, the improvement of scientific and technological level will inhibit the aggravation and diffusion of haze [13,14]. Therefore, *Tec* is expected to be negative for *Pm*.
- Foreign direct investment (*FDI*) is measured by the total amount of foreign direct investment actually utilized, which reflects the economic opening-up of one city [59,60]. After entering WTO in 2001, China has introduced large numbers of foreign investment to promote economic development. *FDI* brought not only many advanced production technologies, but also lots of polluting enterprises that directly lead to the deterioration of China’s air quality [60]. Therefore, the effect of *FDI* on *Pm* remains controversial.

Descriptive statistics of each variable are presented in Table 1. These statistics include abbreviation, mean value, standard deviation, and data sources. The data of core independent variables and control variables are mainly from China City Statistical Yearbook (CCSR), China Statistical Yearbook for Regional Economy (CSYR). For the missing *Us* and *Tec* data, provincial and municipal statistical yearbooks and official websites of corresponding cities between 2002 and 2017 were searched to obtain them. It should be noted that the study area covers 342 prefecture-level cities of China (excluding Taiwan, Hong Kong, Macao, Sansha City, etc.). Due to the adjustment of administrative divisions in some cities, their data were merged according to the division standard of municipal administrative units of China in 2016 [6]. All variables are in logarithmic form.

## 4. Results

### 4.1. Spatiotemporal Distribution Analysis

**Figure 1:**
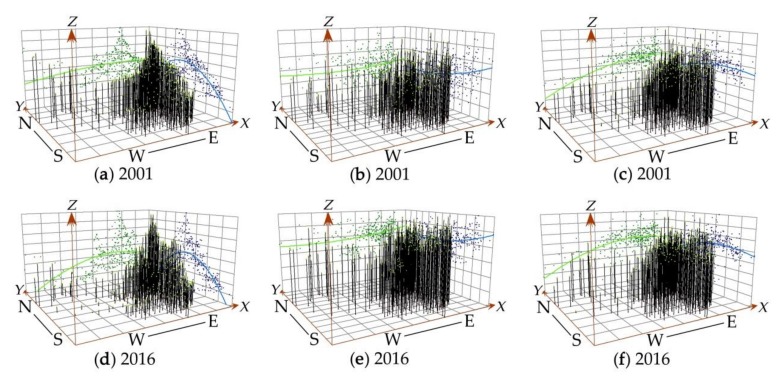
The spatiotemporal patterns of haze pollution in 2001 (**a**) and 2016 (**d**); the spatiotemporal patterns of urban scale in 2001 (**b**) and 2016 (**e**); the spatiotemporal patterns of urban agglomeration in 2001 (**c**) and 2016 (**f**). In these figures, the *X*, *Y*, and *Z* axes represent longitude, latitude, and the attribute values of geographical features (concentration of PM_2.5_, urban scale, or urban agglomeration), respectively. The green and blue points are the projections of the attribute values of geographical features (*Z* values) on the *X*, *Z* and *Y*, *Z* plane, respectively. The green and blue curves represent the fitting second-order polynomials of the scatter plot on each corresponding plane.

In order to represent the spatial characteristics and evolution trend of haze pollution, urban scale, and agglomeration in China from 2001 to 2016, the three-dimensional (3D) trend analysis of ESDA was used to conduct spatial visualization of their data in 2001 and 2016. As shown in Figure 1, haze pollution, urban scale, and agglomeration in China all have a spatial distribution pattern of high in the east (E) and low in the west (W) [30]. However, their spatial distributions are significantly different in the south (S) and north (N) direction, with the former high in the north and low in the south, while the latter two showing exactly the opposite distribution pattern [37]. In addition, the evolution of China’s urban population size and economic agglomeration shows a relatively stable trend from 2001 to 2016.

### 4.2. Spatiotemporal Correlation Analysis

The spatiotemporal correlation of haze pollution with urban scale and agglomeration can be revealed by measuring their Moran’s I. Firstly, the spatial distribution characteristics of haze pollution, urban scale, and agglomeration in 342 Chinese cities during 2001 to 2016 were revealed by their Moran’s I values. Secondly, the bivariate Moran′s I values of urban haze, urban scale, and agglomeration and their significance were calculated to explore their overall spatial correlation characteristics by the Formula (1).

#### 4.2.1. Global Spatiotemporal Correlation

**Figure 2:**
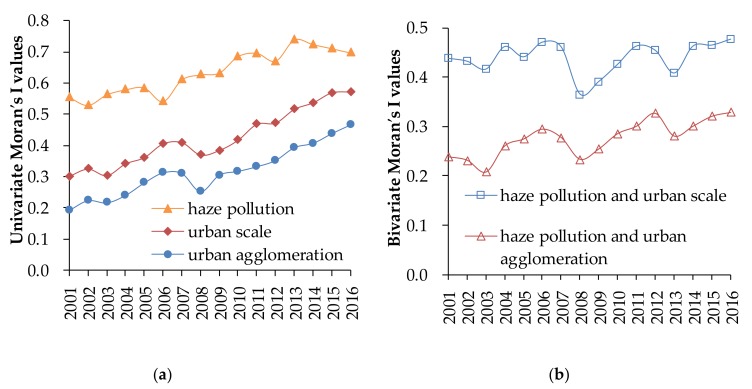
The Univariate Moran’s I values of haze pollution, urban scale, and agglomeration (**a**) and the bivariate Moran’s I values of them (**b**).

Haze pollution, urban scale, and agglomeration of Chinese cities show significantly positive spatial autocorrelation with their Moran′s I values all passing the significance test of 0.05. As is shown in Figure 2a, the univariate Moran′s I values of haze pollution fluctuated between 0.529 and 0.739 during 2001 to 2016 and showed a downward trend after 2013. This indicates that cities in China with similar degrees of haze pollution tended to be closer to each other in space during the study period. The univariate Moran′s I values of urban scale and urban agglomeration fluctuated, increasing from 0.301 and 0.191 in 2001 to 0.572 and 0.465 in 2016, respectively, indicating their spatial dependences in Chinese cities showed a significant upward trend. The univariate Moran’s I values decreased obviously in 2008, which may be related to the decline of population urbanization and economic agglomeration in China caused by the global financial crisis [6,37].

There existed significant spatial correlations between haze pollution and urban scale and agglomeration, with their correlation degrees showing a W-shaped upward trends. From the Figure 2b, the bivariate Moran′s I values of haze pollution, urban scale, and agglomeration are significantly positive, showing that haze pollution is highly spatially correlated with the population size and distribution of economic agglomeration. The bivariate Moran′s I values showed a W-shaped fluctuating upward trend, among which the fluctuation level of haze pollution and urban scale was higher than that of haze pollution and urban agglomeration.

#### 4.2.2. Local Spatiotemporal Correlation

In order to further explore the local spatiotemporal correlation of haze pollution and urban scale and agglomeration in Chinese cities, the results in 2001, 2008, and 2016 were selected to visualize the local spatial correlation characteristics, which were divided into five types of LL, HH, LH, HL, and No (no significant). The first four types are spatially significant, while the correlation of No-type cities is not spatially significant. On the whole, the number of cities with positive spatial correlation (HH and LL types) between haze pollution and urban scale and agglomeration increased the most during 2001 to 2016, mainly located in mid-eastern China [6]. This indicates that the expansion of urban population size and the increase of economic agglomeration in China may have led to the expansion of the spatial scope of haze pollution.

**Figure 3:**
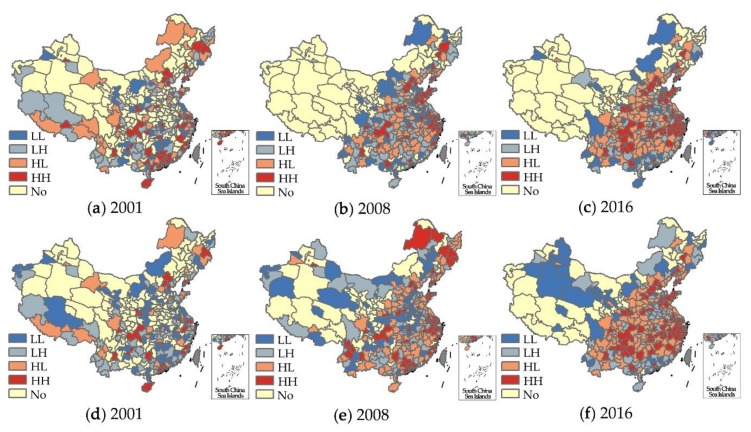
The Bivariate LISA clustering of urban scale and haze pollution in 2001 (**a**), 2008 (**b**), and 2016 (**c**); The Bivariate LISA clustering of urban agglomeration and haze pollution in 2001 (**d**), 2008 (**e**), and 2016 (**f**).

Specifically, the number of LL (low urban scale–low haze pollution) and HH (high urban scale–high haze pollution) type cities was increasing, and the spatial scope of positive impact of urban scale on haze pollution was gradually expanding. The number of LL- and HH-type cities was 87 in 2001, 95 in 2008, and 104 in 2016, accounting for 46.52%, 37.85%, and 39.39% of the significant cities, respectively. From Figure 3a–c, the cities of HH (high urban scale–high haze pollution) type were mainly located in Beijing–Tianjin–Hebei, Shandong Peninsula, Yangtze River Delta, and Chengyu urban agglomeration. This shows that the scale effects of Chinese urban agglomerations have brought negative environmental externalities [14,33,37]. The cities of HL (high urban scale–low haze pollution) type with larger population and better air quality were mainly resource-based cities or distributed at the edge of these urban agglomerations above, which were affected by the spillover effect of central cities’ population size [34,54]. Most of the cities of LL (low urban scale–low haze pollution) type are the third-tier and fourth-tier cities of China with better ecological environment. The number of LH (low scale–high haze) cities is relatively small, most of which are resource-based cities or fringe cities of urban agglomeration with heavy haze pollution.

The number of HH (high urban agglomeration–high haze pollution)-type cities was 32 in 2001, 57 in 2008, and 67 in 2016, respectively. From Figure 3d–f, the cities of HH type were also mainly distributed in those urban agglomerations above and their surrounding areas. This indicates higher urban economic agglomeration will have a greater negative impact on air quality. During the study period, the number of LL-type cities dropped from 60 to 45, which were mainly distributed in south and northwest China with spatial locking effect. The cities of HL type had the largest number, and were mainly distributed in mid-eastern. The cities of LH type were mainly located in central plains urban agglomeration and southern provinces (Guangdong, Guangxi, Yunnan, and Fujian). The economic development of these regions was relatively unbalanced, whose air quality had been frequently affected by the diffusion and spread of haze in the past few years [30,44].

### 4.3. Spatial Effects Analysis

#### 4.3.1. Model Estimation

The spatial autocorrelation test shows that haze pollution in China is obviously spatially dependent under the influence of population size and economic agglomeration. Therefore, the spatial effect of haze pollution among different cities cannot be neglected. Firstly, SEM and SLM were compared by (Robust) LM test. LM lag, Robust LM lag, LM error, and Robust LM error of the proposed model all passed the significance test of 0.01, indicating the spatial dependence of the haze pollution interpretation model of Chinese cities has not only spatial lag term but also spatial error term. Secondly, SDM was established to determine whether it can be simplified to SEM and SLM through Walds and LR tests [6,46]. The test results of Wald spatial error, Wald spatial lag, LR spatial error, and LR spatial lag also passed the significance test of 0.01. Therefore, SDM of explaining haze pollution cannot be simplified to SLM or SEM. Finally, Hausman test results rejected random effects at the significance test of 0.05 level and accepted SDM with fixed effects. Thus, SDM with no fixed effects (NoF), time fixed effects (TF), spatial fixed effects (SF) and spatial-temporal fixed effects (STF) were selected for parameter estimation in our study. In the regression results (Table 2), the values of Log L and *adj*.*R^2^* of the SF column (the fourth column) are larger than that of the NoF, TF, and STF columns. Therefore, the SDM with SF is the best model to explain the spatial effect of urban scale and agglomeration on haze pollution of Chinese cities.

#### 4.3.2. Parameter Estimation Results

From Table 2, the spatial spillover coefficient *δ* is 0.416 and significant at the 0.01 level in the fifth column of the left half of the table, indicating that there are significant spatial spillover effects of haze pollution among Chinese cities. The elasticity coefficients of urban scale (ln*Us*) and urban agglomeration (ln*Ua*) to haze pollution are 0.296 and 0.205, respectively, and both of which pass the significance test of 0.05. This indicates that the expansion of urban population size and the improvement of economic agglomeration are important factors leading to the aggravation of haze pollution. In addition, the effect of urban scale on haze is stronger than that of urban agglomeration, mainly due to the more diverse and complex impacts of urban population growth aggravating the haze concentration [37,55]. The elasticity coefficients of ln*Us* and ln*Ua* with spatial lag are 0.107 and 0.293, respectively, showing that the urban population size and economic agglomeration of neighboring cities are also important factors leading to the aggravation of haze in the local city. The impact of economic agglomeration of neighboring cities on haze in the local city is stronger than that of the population size. Therefore, urban economic agglomeration will bring about stronger spatial spillover effects on haze pollution.

Further analysis of the influence of control variables on haze pollution shows that the coefficients of ln*Pgdp*, ln*Str*, ln*Tec*, and ln*FDI* are all significant at 0.10 level, and their elasticities to haze pollution are 0.135, −0.161, −0.132, and 0.149, respectively (Table 2). *Pgdp* and *FDI* have a certain negative impact on air quality, while improvement of *Str* and *Tec* will significantly reduce *Pm*. The elasticity coefficients of W *ln*Pgdp*, W *ln*Str*, W *ln*Tec* are 0.076, −0.136, and −0.171 and significant at 0.10 level, indicating that the improvement of the economic development level in neighboring cities will have a certain impact on the increase of haze pollution in local city through spatial spillover effect. However, the optimization of industrial structure and improvement of science and technology level in neighboring cities will significantly reduce the PM_2.5_ concentration in local city. The elasticity coefficient of W*ln*FDI* is positive but not significant, which means that it is uncertain whether the increase of *FDI* in neighboring cities will affect the haze in local city [59,60].

#### 4.3.3. Spatial Effect Decomposition

By decomposing the spatial effect of haze pollution into direct and indirect effects, we obtained Table 3. The direct effect coefficients of ln*Us* and ln*Ua* are 0.238 and 0.117, respectively, which reflects that every 1% of positive change of China’s urban population scale and economic agglomeration will directly lead to an elastic increase of haze pollution by 0.238% and 0.117%, respectively. The indirect effect coefficients of ln*Us* and ln*Ua* are 0.161 and 0.204, showing haze pollution in local city will increase by 0.161% and 0.204% for every 1% of positive change of population size and economic agglomeration in neighboring cities. The direct effect of ln*Us* is greater than that of ln*Ua*, while the indirect effect of ln*Ua* is greater than that of ln*Us*. This provides evidence for the asymmetry of their spatial effects on haze pollution.

From Table 3, the direct effect coefficients of ln*Pgdp*, ln*Str*, and ln*Tec* are 0.086, −0.105, and −0.081, respectively. *Pgdp* has a significantly positive spatial effect, indicating that a 1% increase in *Pgdp* will directly lead haze pollution to increase by 0.086%. However, both *Str* and *Tec* have negative spatial effect. Every 1% positive change in *Str* and *Tec* will reduce haze concentration by 0.105% and 0.081%, respectively. The indirect effect coefficients of ln*Pgdp*, ln*Str*, and ln*Tec* are 0.02, −0.093, and −0.101, indicating that every 1% increase of *Pgdp* in neighboring cities will promote the haze in local city to increase by 0.020%. The indirect effects of ln*Str* and ln*Tec* are negative, suggesting that every 1% increase of *Str* and *Tec* in neighboring cities will bring about a decrease of 0.093% and 0.101% of haze pollution in local city. Both the direct and indirect effect coefficients of ln*FDI* are not significant, which indicates that the relationship between *FDI* and *Pm* is still complex. The claim of “pollution heaven” remains to be proven and confirmed [61,62].

## 5. Discussion

Urbanization has not only brought great economic and social benefits, but also brought environmental problems such as haze pollution, which is particularly prominent in the process of rapid urbanization of China, accordingly providing a typical domain and case for the in-depth discussion of their relationship [16,17]. The generation of haze is not only related to its physical and chemical causes, but also related to urban population scale and economic agglomeration, on which the research is relatively little [14,18,19,20]. Scale effect and agglomeration effect are the two basic spatial effects of urban development. Urban scale refers to population size and land size. Due to good continuity of population data and relatively stable statistical caliber, most literature selected urban population scale to represent urban scale [34,51,54]. Urban agglomeration is formed by the concentration of economic activities in urbanization process, mainly reflected by the continuous improvement of GDP. Urban population growth is based on urban economic agglomeration, and urban economic agglomeration requires population growth [34,56]. Urban scale and urban agglomeration complement each other and jointly promote the development of cities [34,37]. After selecting four important factors as control variables (per capita GDP, advanced industrial structure, scientific and technological level, and Foreign direct investment), we focused on the asymmetrical spatial effects of urban scale and agglomeration on haze pollution in China. The reason is that we found the excessively one-sided pursuit of rapid urbanization including population expansion and economic agglomeration in many cities of China and other countries ignored its environmentally negative externalities [19,63]. Therefore, China should summarize the experiences and lessons from high-speed urbanization bringing air pollution in various cities (large, medium, and small cities) to promote the prevention and control of haze and ultimately realize sustainable and healthy development [1,64]. This study supplemented the theoretical perspective of the research on the impact of haze pollution through highlighting the significance of considering asymmetry of influencing factors and their spillover effects among destinations when evaluating the relationship between haze pollution and urban scale and agglomeration.

Through spatial distribution analysis, the features of spatial differentiation and couplings and coordination of haze pollution, urban scale, and agglomeration in Chinese cities are found by 3D trend analysis. This finding indicates that the regional imbalance of geographical elements has brought challenges to environmental governance for different cities, and differentiated haze countermeasures will be more necessary and meaningful [37]. Furthermore, the results of spatial autocorrelation analysis suggest haze pollution, urban scale, and agglomeration in Chinese cities have not only significantly spatial dependence, but also obviously spatial correlation. Although a large amount of literature holds that China’s urbanization is transforming from scale expansion to quality improvement and the spatial correlations between China’s haze and urban population size and economic agglomeration are showing a decoupling trend [37,56,57,58], the results of our study do not support these findings. The small fluctuations of Moran′s I values in 2008 and after 2013 are mainly due to international special events and the air control measures implemented by local governments of China beginning to show positive effects [6,10,22].

In addition, from the perspective of spatial effects, our work paper quantitatively confirms the asymmetrical spatial effects of urban scale and agglomeration on haze pollution by using a spatial Durbin model with spatial fixed effects, which could deepen the understanding of the relationship between haze pollution and urban scale and agglomeration and provide a method reference for follow-up research. The SDM with spatial fixed effects could also be more broadly used in haze impact studies, as well as studies on the influence of other geographical factors. We also hope our work will provide references for quantitatively evaluating the effects of urban scale and agglomeration on haze in Southeast Asia, where the air pollution is also prominent these years [41]. Due to the haze pollution not being a localized problem, our findings have major implications for policy-makers to achieve sustainable urbanization. For example, the existences of spatial spillover effect and spatial asymmetries of urban scale and agglomeration on haze pollution require them to strengthen intergovernmental environmental management [6,14]. Finally, the effect of urban scale on haze is stronger than that of urban agglomeration, mainly due to the more diverse and complex impacts of urban population growth. The growth of urban population has brought the increase of private car travel, the expansion of urban construction land, and the increase of housing demand and consumption, which aggravate haze pollution and reduce the air quality [36,38]. However, urban economic agglomeration will bring about stronger spatial spillover effects on haze pollution. In the process of economic development, cities’ industries that affect the environment are often located in its fringe areas or the areas adjacent to other cities [40]. This industrial layout pattern has a great negative impact on the environmental quality of the neighboring cities [58]. The heterogeneity of determinants revealed by our work will help local governments to better understand the influencing factors of haze and put forward more targeted development strategies.

## 6. Conclusions and Policy Implications

### 6.1. Conclusions

Taking 342 Chinese cities from 2001 to 2016 as an example, ESDA and SDM were adopted to analyze the asymmetrical spatial effects of urban scale and agglomeration on haze pollution and inter-city spatial spillover effect. The following main conclusions were drawn:

(1) In the 21st century, the spatial pattern of urban population size and economic agglomeration brought by China’s rapid urbanization has certain regional overlap with the distribution of haze. From 2001 to 2016, the spatial distribution patterns of urban scale, agglomeration, and haze in China all presented an asymmetric spatial distribution pattern of “high in the east and low in the west”, which was opposite to that of urban scale and agglomeration, with the center of Chinese urbanization having been moving southward. These regional overlap differences show that the distribution of haze is not only affected by urban scale and agglomeration, but also impacted by regional natural conditions, climate change, industrial structure, technological levels and so forth.
(2) The local spatial correlation of haze pollution with urban scale and agglomeration is more obvious on the scale of China’s urban agglomerations. The spatial distribution of haze pollution in Chinese cities is affected by the effect of urban agglomeration. The results of their univariate and bivariate Moran’s I show a significantly positive global spatial autocorrelation in China, with the levels of spatial dependence fluctuating upward. The local spatial correlation of haze pollution and urban scale and agglomeration was dominated by HH and LL types, the number of which increased continuously during 2001 to 2016. The cities of HH type were mainly distributed in Shandong Peninsula, Beijing-Tianjin-Hebei, Yangtze River Delta, and Chengyu urban agglomeration.
(3) Haze pollution in neighboring cities could interact with each other, and has significant spatial spillover effect with the coefficient of 0.416. At the same time, both urban scale and agglomeration have an important impact on haze pollution. The direct effect of urban scale on haze pollution is more prominent than that of urban agglomeration, while the indirect effect of urban agglomeration in neighboring cities is more obvious than that of urban scale in local city. Every 1% positive change of urban scale and agglomeration in Chinese cities will promote PM_2.5_ concentration to increase by 0.238% and 0.117%, respectively. However, the spatial interaction of urban scale and agglomeration among cities will indirectly lead haze to increase by 0.161% and 0.204% in neighboring cities.
(4) Decomposing the spatial effect of control variables (per capita GDP, advanced industrial structure, scientific and technological level, foreign direct investment) into direct and indirect effects, we found that the improvement of per capita GDP will increase haze pollution in both local and neighboring cities, while the improvement of advanced industrial structure and scientific and technological level will alleviate haze pollution in local city and neighboring cities, among which the determinant of advanced industrial structure is more prominent in reducing haze in local city, and the determinant of scientific and technological level is more obvious in reducing haze in neighboring cities. Foreign direct investment, which plays an important role in the rapid development of urbanization in China, has no clear relationship with haze pollution.

At the critical stage that China’s urbanization transforms from quantitative expansion to quality improvement, it is unwise for Chinese cities to blindly copy the existing western urbanization theories [37]. It will be essential for policy-makers to fully understand healthy urbanization in developed countries like Europe and America [1,65]. Haze pollution is a regional and complex problem rather than a simple problem within one city. Therefore, only by investigating more comprehensive factors (not only socioeconomic factors) of haze pollution can we make a more scientific judgment and understanding on it to achieve healthy and sustainable urbanization [65]. This study has several limitations. Firstly, the expansion of urban population and economic agglomeration is a rather complex process involving nature, economy, society, technology, and management system, and a more comprehensive index system of control variables will be established by us in future work [14]. Secondly, this study conducts a realistic study mainly based on statistical data, the application of big data (such as traffic big data, industrial big data, Point of Interest (POI), PM_2.5_ data-based monitoring stations at every moment, and so on) to more accurately characterize urban scale, agglomeration, and haze pollution will be welcomed in the future. Thirdly, to measure the spatial spillover effects of urban scale and agglomeration on haze pollution more specifically and in more detail, the alternative ways (the inverse distance-based spatial weights matrix, economic-based weights matrix, and nested weights matrix) to construct spatial weight matrix in our future work are meaningful. Finally, a longer period of sample data, qualitative, and quantitative methods will be selected to more thoroughly and accurately explore and further deepen the understanding of haze pollution, urban population size, economic agglomeration, as well as their interaction mechanism in the follow-up studies.

### 6.2. Policy Implications

Based on this study and extensive consultations, we put forward a wide range of program and policy recommendations that will help improve air quality despite the existence of emerging sources of air pollution and challenges to natural resources management. The recommendations of our study for policy-makers are explained as follows:

(1) Nowadays, China should abandon the development mode of land- and economy-centered urbanization and choose the way forward for people- and environment-oriented urbanization. Inclusive growth and a green economy should be the government’s guiding principles in the future [51,54]. To support these principles, China needs to restructure its economic and industrial structure to reflect environmental externality, expand the use of market-based instruments to control air pollution, and carry out legal reforms to clarify responsibility of each interest subject and promote inter-enterprise and inter-governmental cooperation [66,67].
(2) The expansion of urban scale and economic development of Chinese cities has caused seriously negative impact on air quality that related to the physical and mental health of residents. Local governments must attach great importance to the urgent situation of haze control, strengthen the prevention and governance of haze pollution, and immediately take haze countermeasures of regional joint prevention and control, joint management among departments, and active participation of social forces to improve regional air quality [6].
(3) In the pursuit of population-scale economy, local governments should pay more attention to the improvement of personnel quality and improve the public’s awareness of environmental protection [68]. Moreover, it will be wise and meaningful for local governments to consciously practice the idea of green development and advocate the use of clean energy in production and life. Only in this way can they reduce air pollution caused by the high concentration of population and realize the healthy and sustainable green development of these cities with highly concentrated populations [69].
(4) While pursuing high-speed economic growth, local governments should pay more attention to the upgrading and rationalization of industrial structure and promote the synchronous and balanced development of regional industry and environmental quality. Policy-makers of each city should continue to strengthen the optimization and upgrading of industrial structure according to its own economic development level and resource advantages [58]. In addition, much attention should be paid to the improvement of investment structure, especially the investment structure of science and technology, and advocate the development of green GDP [51]. The efficiency of energy and resource utilization in the production activities of enterprises should also be improved to achieve energy conservation and emission reduction, and offset the environmental pressure brought by the improvement of industrial agglomeration.

## Figures and Tables

**Table 1:** Descriptive Statistics of Variables.

Variables Type	Variable Full Name (Unit)	Abbreviation	Mean	Standard Deviation	Data Sources
Dependent variable	Particulate Matter (PM_2.5_) concentration (μg/m^3^)	*Pm*	0.81	1.02	SEDAC
Core independent variables	Urban scale (ten thousands)	*Us*	5.62	8.95	CCYS, CSYR
	Urban agglomeration (ten thousands)	*Ua*	3.22	5.60	CCYS
Control variables	Per capita GDP (yuan)	*Pgdp*	4.55	3.20	CCYS, CSYR
	Advanced industrial structure (%)	*Str*	0.23	0.09	CCYS
	λ Scientific and technological level (%)	*Tec*	0.18	0.07	CCYS, CSYR
	Foreign direct investment (ten thousands)	*FDI*	6.35	2.15	CCYS

**Table 2:** SDM results for urban scale, agglomeration, and haze pollution in China.

Variables	NoF	TF	SF	STF	Variables	NoF	TF	SF	STF
ln*Us*	0.191 *** (0.06)	0.253 *** (0.09)	0.296 *** (0.27)	0.172 ** (0.18)	W *ln*Us*	0.098 (0.01)	0.110 ** (0.17)	0.107 * (0.28)	0.165 * (0.66)
ln*Ua*	0.124 * (0.09)	0.179 ** (0.01)	0.205 *** (0.18)	0.246 *** (0.52)	W *ln*Ua*	0.112 * (0.07)	0.199 *** (0.15)	0.293 ** (0.60)	0.090 ** (0.01)
ln*Pgdp*	0.117 *** (0.10)	0.102 ** (0.16)	0.135 * (0.21)	0.147 ** (0.45)	W *ln*Pgdp*	0.103 ** (0.24)	0.022 * (0.01)	0.076 * (0.08)	0.100 *** (0.26)
ln*Str*	−0.082 (−0.03)	−0.079 (−0.01)	−0.161 * (−0.00)	−0.102 * (−0.11)	W *ln*Str*	−0.076 ** (−0.12)	0.210 * (0.16)	−0.136 ** (−0.02)	0.109 (0.05)
ln*Tec*	−0.242 * (−0.83)	−0.112 ** (−0.31)	−0.132 ** (−0.01)	−0.076 * (−0.24)	W *ln*Tec*	−0.110 * (−0.77)	−0.002 * (−0.08)	−0.171 * (−0.12)	−0.069 (−0.04)
ln*FDI*	0.152 * (0.14)	0.172 (0.07)	0.149 ** (0.15)	0.121 (0.13)	W *ln*FDI*	0.108 ** (0.09)	0.101 ** (0.05)	0.125 (0.97)	0.118 ** (0.27)
*Adj.R^2^*	0.616	0.831	0.857	0.563	*δ*	0.295 * (10.37)	0.287 * (11.03)	0.416 *** (14.12)	0.532 (16.01)
*Log L*	−3822.051	−3478.158	−3342.298	−3412.019					

Note: NoF, TF, SF, and STF represent SDM with no fixed effects, time fixed effects, spatial fixed effects, and spatial-temporal fixed effects, respectively. ***, **, and * indicate significance at the 0.01, 0.05, and 0.10 level, respectively. The number in parentheses is the t-statistic of each coefficient. The same below.

**Table 3:** Direct and indirect effects of urban scale, agglomeration, and haze pollution in China.

Variables	ln*Us*	ln*Ua*	ln*Pgdp*	ln*Str*	ln*Tec*	ln*FDI*
Direct effect	0.238 *	0.117 ***	0.086 *	−0.105 *	−0.081 *	0.156
Indirect effect	0.161 **	0.204 *	0.020 **	−0.093 *	−0.101 **	0.101
Total effect	0.399 *	0.321 **	0.106 *	−0.198 **	−0.182 **	0.257

Note: ***, **, and * indicate significance at the 1%, 5%, and 10% level, respectively. The number in parentheses is the *t*-statistic of each coefficient. The same below.
